# Transplanting Soil Microbiomes Leads to Lasting Effects on Willow Growth, but not on the Rhizosphere Microbiome

**DOI:** 10.3389/fmicb.2015.01436

**Published:** 2015-12-21

**Authors:** Etienne Yergeau, Terrence H. Bell, Julie Champagne, Christine Maynard, Stacie Tardif, Julien Tremblay, Charles W. Greer

**Affiliations:** ^1^Energy Mining and Environment, National Research Council CanadaMontreal, QC, Canada; ^2^Biodiversity Centre, Institut de Recherche en Biologie Végétale, Université de Montréal and Jardin Botanique de MontréalMontréal, QC, Canada

**Keywords:** willow, microbiome engineering, phytoremediation, microbiome transplantation, contaminated soils

## Abstract

Plants interact closely with microbes, which are partly responsible for plant growth, health, and adaptation to stressful environments. Engineering the plant-associated microbiome could improve plant survival and performance in stressful environments such as contaminated soils. Here, willow cuttings were planted into highly petroleum-contaminated soils that had been gamma-irradiated and subjected to one of four treatments: inoculation with rhizosphere soil from a willow that grew well (LA) or sub-optimally (SM) in highly contaminated soils or with bulk soil in which the planted willow had died (DE) or no inoculation (CO). Samples were taken from the starting inoculum, at the beginning of the experiment (T0) and after 100 days of growth (TF). Short hypervariable regions of archaeal/bacterial 16S rRNA genes and the fungal ITS region were amplified from soil DNA extracts and sequenced on the Illumina MiSeq. Willow growth was monitored throughout the experiment, and plant biomass was measured at TF. CO willows were significantly smaller throughout the experiment, while DE willows were the largest at TF. Microbiomes of different treatments were divergent at T0, but for most samples, had converged on highly similar communities by TF. Willow biomass was more strongly linked to overall microbial community structure at T0 than to microbial community structure at TF, and the relative abundance of many genera at T0 was significantly correlated to final willow root and shoot biomass. Although microbial communities had mostly converged at TF, lasting differences in willow growth were observed, probably linked to differences in T0 microbial communities.

## Introduction

Microorganisms colonize all plant components, and plants interact constantly with this complex microbiome. Between 5 and 20% of a plant's photosynthetic yield is transferred to its microbiome, and this occurs mainly through the roots (Marschner, 1995). As a result of this transfer, the rhizosphere supports much higher bacterial abundance and activity, not only when compared to other plant compartments, but also relative to bulk soil (Smalla et al., 2001; Kowalchuk et al., 2002). However, bacterial diversity in the rhizosphere is generally lower than is observed in bulk soil (Marilley and Aragno, 1999) while microbial community composition is distinct (Smalla et al., 2001; Kowalchuk et al., 2002; Griffiths et al., 2006; Kielak et al., 2008; Bulgarelli et al., 2012; Peiffer et al., 2013), suggesting a strongly selective environment in the rhizosphere. This selection pressure often varies between plant species (Haichar et al., 2008; Berg and Smalla, 2009) and even genotypes (Lundberg et al., 2012; Sugiyama et al., 2012). This selection pressure results from the exudation of specialized antimicrobials (e.g., flavonoids, salicylic acid, phytoalexins), or compounds that provide carbon (e.g., organic acids, aromatic compounds) and/or nitrogen (e.g., amino acids) to microbes (Badri et al., 2009). An emerging view in the microbial ecology of microbe–host systems is that the host and its microbial inhabitants are an inseparable entity, and actually function as a meta-organism or a holobiont (Bosch and McFall-Ngai, 2011; Vandenkoornhuyse et al., 2015). Interactions between plants and microbes have evolved over millions of years, and these relationships allow the plant–microbe meta-organism to minimize overall stress by, among other mechanisms, deterring pathogens (St-Arnaud and Vujanovic, 2007; Sikes et al., 2009; Mendes et al., 2011), increasing N and P uptake (Richardson et al., 2009), protecting against abiotic stress (Marasco et al., 2012; Selvakumar et al., 2012), and detoxifying the environment (Siciliano et al., 2001). Because of these intricate links, engineering of the plant host without considering the microbiome likely limits the phenotypic optimum that can be achieved (Bell et al., 2014b; El Amrani et al., 2015; Quiza et al., 2015).

Depending on its composition and activity, the plant microbiome can be either beneficial or deleterious to plant health, and shifting this delicate balance has huge implications for plant productivity. Several authors have suggested that optimizing the plant microbiome is a possible solution to the shortage of food on the planet (Morrissey et al., 2004; Glick, 2014). Manipulating the plant microbiome has the potential to reduce the incidence of plant disease (Andrews, 1992; Bloemberg and Lugtenberg, 2001), increase agricultural production (Bakker et al., 2012), reduce the need for chemical inputs (Adesemoye et al., 2009), reduce greenhouse gas emissions (Singh et al., 2010), and increase plant-mediated removal of pollutants (Bell et al., 2014b). One approach to soil microbiome engineering is the use of blanket treatments (e.g., fertilization) to stimulate the whole microbial community, but this may lead to the stimulation of microbes that do not optimally perform targeted functions (Bell et al., 2011). Another is to introduce microorganisms to soil that are capable of performing the desired functions (i.e., bioaugmentation), like polychlorinated biphenyl- (Secher et al., 2013), polycyclic aromatic hydrocarbon- (Baneshi et al., 2014), and diesel- (Chuluun et al., 2014) degradation. However, the abundance and functional diversity of indigenous soil microbes allows them to occupy most available ecological niches, and so attempts to introduce new microorganisms have been met with limited success (Thompson et al., 2005; Gerhardt et al., 2009). Instead, disrupting microbial communities by removing specific taxonomic groups or reducing the overall microbial load may open niches for microbial colonization. Specific inhibitors like antibiotics and fungicides have been used to disrupt soil microbial communities and promote specific functions of interest (Bell et al., 2013; Qiu et al., 2014). For instance, Bell et al. (2013) used two antibiotics to inhibit specific microbial groups in diesel-contaminated soils, and found that using the two antibiotics in combination in nutrient-amended soils resulted in higher diesel degradation rates than controls or soils treated with only one antibiotic. In another study, Qiu et al. (2014) used fungicides in the rhizosphere of cucumber, which resulted in a higher incidence of disease when a pathogen was inoculated, but reduced disease incidence and increased plant growth when the pathogen was inoculated along with an antagonist bacteria. Although, the feasibility and ethics of using such approaches for modifying soil microbiomes in the field is debatable, these studies suggest potential mechanisms by which complex microbiomes can be modified. Other studies demonstrated that inoculation with microbial consortia was a more effective approach than single strain inoculation, as microorganisms appear to work synergistically to efficiently degrade petroleum hydrocarbon contaminants (Alarcón et al., 2008; Afzal et al., 2012). Further factors complicating efforts to engineer plant microbiomes include differences in the physiology and ecology of soil inhabitants, resulting in differential responses of bacterial and fungal activity, growth, and diversity to key rhizosphere parameters like pH (Rousk et al., 2009, 2010) and plant identity (Haichar et al., 2008; Berg and Smalla, 2009).

Willows (*Salix* spp.) have been used as model plants for phytoremediation, as they rapidly produce high amounts of biomass, including an extensive root system capable of stimulating soil microbial communities. One of the keys to effective phytoremediation with willows is the optimization of growth, biomass production, and survival in highly contaminated environments. The goal of the present study was to observe whether a complex microbiome could be transferred from one plant to another, and whether this also transferred certain characteristics of the original plant (growth, biomass production, and survival in a stressful environment). In other words, how much of the plant phenotype is related to the root-associated microbiome? Clonal willow clippings were planted for two generations in soil originating from a hydrocarbon-contaminated field site. First generation willows were planted into the unmodified soil, and soils associated with willows that showed dramatically different growth characteristics were harvested and used to inoculate gamma-irradiated soil from the same site. A second generation of willows was planted into these inoculated soils. We hypothesized that inoculation with the rhizosphere soil of large first-generation willows would result in larger second-generation willows with lower mortality than when inoculating with soil associated with smaller or dying first-generation plants.

## Materials and methods

### Soil inoculum

Soil was retrieved from an experiment in which clonal willows (*Salix purpurea* “Fish Creek”) were planted into a homogenized highly petroleum-contaminated soil (C10–C50 concentration: 17,500 mg/kg). Most of the introduced willows died; out of 100 initial plants, only 11 were alive after 173 days. The rhizosphere of a large surviving willow (height of 128 cm, shoot fresh weight of 62.00 g, used to inoculate the LA treatment), the rhizosphere of a small surviving willow (height of 80 cm, shoot fresh weight of 43.46 g, used to inoculate the SM treatment) and the bulk soil from a pot in which the willow had died (used to inoculate the DE treatment), were harvested on 21 October 2013 by collecting the soil that remained attached to the root system after vigorously shaking the willows (for the rhizosphere) or by taking a surface soil sample in the middle of the pot (for the bulk soil). These soils represent the three different soil inocula used in subsequent experiments. Soils were transported at 4°C and frozen at −20°C until used for downstream steps.

### Experimental design

Fresh soil was collected at the site of a former petrochemical plant in Varennes, Quebec, Canada, within 2 m of the excavation site of the contaminated soil described above. Soil was mixed thoroughly, transferred in 20 L pails and sent to Nordion (Laval, Quebec, Canada) for gamma irradiation at a dose of 50 kGy, to disrupt the microbiome and minimize the soil microbial load. Following a previous identical irradiation of the same soil, no cultivable microorganisms could be retrieved from the soil (T.H. Bell, unpublished observations), even though bacterial, archaeal, and fungal DNA could be amplified. For each treatment type (DE, LA, and SM treatments), ~10 kg of irradiated soil was mixed with 1 kg of the different soil inocula and distributed into ten 1 L pots. A control treatment was left uninoculated (CO treatment), resulting in four treatments with 10 replicate pots each. A willow clipping (*S. purpurea* “Fish Creek”) was planted to a depth of 10 cm in the middle of each pot. Willow clippings were also planted in 10 pots filled with potting soil to evaluate willow growth under ideal conditions. Pots were placed in a greenhouse (February 18, 2014), and were incubated at a temperature of 20°C during the day and 18°C overnight. High-pressure sodium lamps (430 W) were illuminated for 18 h a day, starting at 6:00 a.m. The position of the pots on the greenhouse bench was determined using a random number generator.

### Sampling

Each inoculum type (three samples) was sampled before mixing with irradiated soil and soils from the pots were sampled before planting with willow clippings (February 17, 2014, 4 treatments × 10 replicates = 40 samples at T0). Willow growth was measured on March 21, 2014 (32 days after planting), on April 17, 2014 (59 days after planting), and on May 23, 2014 (95 days after planting). Rhizosphere soils were sampled at the end of the experiment (May 28, 2014, after 100 days, 4 treatments × 10 replicates = 40 samples at TF) by collecting the soil that remained attached to the root system after vigorously shaking the willows. Willow roots and shoots (excluding the original willow cuttings) were also harvested at the end of the experiment, dried at 105°C overnight, and weighed.

### DNA extraction, amplification, and sequencing

**Figure 1:**
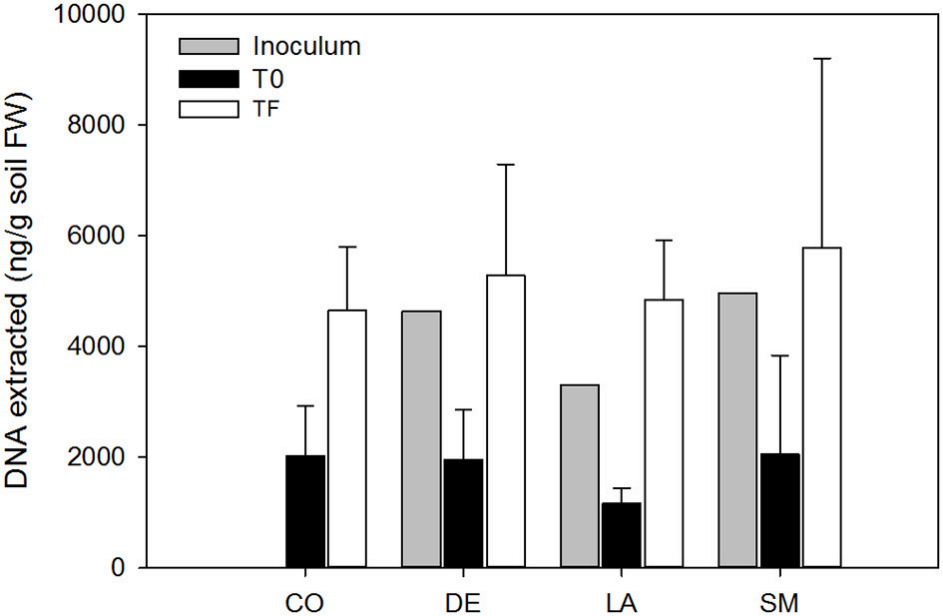
**DNA yields from bulk and willow rhizosphere soil samples taken at T0 and TF (after 100 days) for CO, DE, SM, and LA treatments**. Error bars represent standard deviation.

For the 83 samples, (3 inocula, 4 treatments × 10 replicates at T0 and 4 treatments × 10 replicates at TF), DNA was extracted from an average of 0.352 g of soil using the MoBio Power Soil DNA extraction kit resulting in an average of 1.80 μg of DNA per g of soil at T0 and 5.14 μg of DNA per g of soil at TF (Figure 1). Libraries for sequencing were prepared according to Illumina's “16S Metagenomic Sequencing Library Preparation” guide (Part # 15044223 Rev. B), with the exception of using Qiagen HotStar MasterMix for the first PCR (“amplicon PCR”) and halving reagents volumes for the second PCR (“index PCR”). The template specific primers were (without the overhang adapter sequence): (1) Archaea 16S rRNA gene: Arch516F (5′-TGYCAGCCGCCGCGGTAAHACCVGC-3′) and A806R (5′-GGACTACVSGGGTATCTAAT-3′), (2) bacterial 16S rRNA gene: F343 (5′-TACGGRAGGCAGCAG-3′) and R803 (5′-CTACCAGGGTATCTAATCC-3′), and (3) fungal internal transcribed spacer (ITS): ITS1F (5′-CTTGGTCATTTAGAGGAAGTAA-3′) and 5.8A2R (5′-CTGCGTTCTTCATCGAT-3′). The first PCR (“amplicon PCR”) was carried out for 30 (bacterial 16S) or 35 (archaeal 16S and fungal ITS) cycles with annealing temperatures of 55°C (archaeal 16S and bacterial 16S) or of 45°C (fungal ITS). Diluted pooled samples were then loaded on an Illumina MiSeq and sequenced using a 300-cycles MiSeq Reagent Kit v2 (Archaeal 16S) or a 600-cycles MiSeq Reagent Kit v3 (bacterial 16S and fungal ITS).

### Sequence data treatment

Sequences were analyzed through our internal rRNA short amplicon analysis pipeline (Tremblay et al., 2015). Common sequence contaminants (i.e., Illumina adapters and PhiX spike-in reads) were first removed from raw sequences using a kmer matching tool (DUK; http://duk.sourceforge.net/). Filtered reads were assembled with the FLASH software (Magoč and Salzberg, 2011). Using in-house Perl scripts, assembled amplicons were then trimmed to remove forward and reverse primer sequences that might be included in some reads. Paired-end assembled amplicons were then filtered for quality: sequences having more than 1 N, an average quality score lower than 30, or more than 10 nucleotides having a quality score lower than 10 were rejected.

OTU generation was done using a three step clustering pipeline. Briefly, quality controlled sequences were dereplicated at 100% identity. These 100% identity clustered reads were then denoized at 99% identity using USEARCH (Edgar, 2010). Clusters of less than three reads were discarded and remaining clusters were scanned for chimeras using UCHIME *de novo* followed by UCHIME reference using the Broad's Institute 16S rRNA Gold reference database. Remaining clusters were clustered at 97% identity (USEARCH) to produce OTUs; data were then rarefied to 1000 reads.

Taxonomy assignment of resulting bacterial and archaeal OTUs was performed using the RDP classifier with a modified Greengenes training set built from a concatenation of the Greengenes database (version 13_5 maintained by Second Genome), Silva eukaryotes 18S r118 and a selection of chloroplast and mitochondrial rRNA sequences. ITS organisms were classified using the ITS Unite database (version: sh_qiime_release_13.05.2014). Hierarchical tree files were generated with in-house Perl scripts and used to generate training sets using the RDP classifier (v2.5) training set generator's functionality (Wang et al., 2007). With taxonomic lineages in hand, OTU tables were generated and rarefied to 1000 reads. These OTU tables were used for downstream analysis.

Diversity metrics were obtained by aligning OTU sequences on a Greengenes core reference alignment (DeSantis et al., 2006) using the PyNAST aligner (Caporaso et al., 2010). Alignments were filtered to keep only the V4, V7–V8, or V6–V8 part of the alignment. A phylogenetic tree was built from alignment with FastTree (Price et al., 2010). Alpha (observed species) and beta (weighted or unweighted UniFrac and Bray–Curtis distances) diversity metrics and taxonomic classifications were computed using the QIIME software suite (Caporaso et al., 2010; Kuczynski et al., 2010).

### Statistical analyses

All statistical analyses were carried out in R v3.0.2 (R Core Team, 2013). Analysis of variance (ANOVA) and repeated-measures ANOVA was performed using the “aov” function, Spearman rank-order correlation analyses were performed using the “cor.test” function, Permanova was performed using the “adonis” function of the vegan library, and principal coordinate analyses were performed using the “cmdscale” function of the vegan library based on the Bray–Curtis distance calculated from the OTU matrix using the “vegdist” function of the vegan library. Since biomass could only be measured at TF, correlations and permanova analyses were carried out against the relative abundance of genera at TF, but also at T0 to evaluate whether the relative abundance of a genus, the overall community structure, or microbial diversity at T0 could be related to willow growth at TF.

### Data deposition

Raw sequence data produced in this study was deposited in NCBI under the BioProject accession PRJNA301462.

## Results

### Willow growth and soil DNA yields

**Figure 2:**
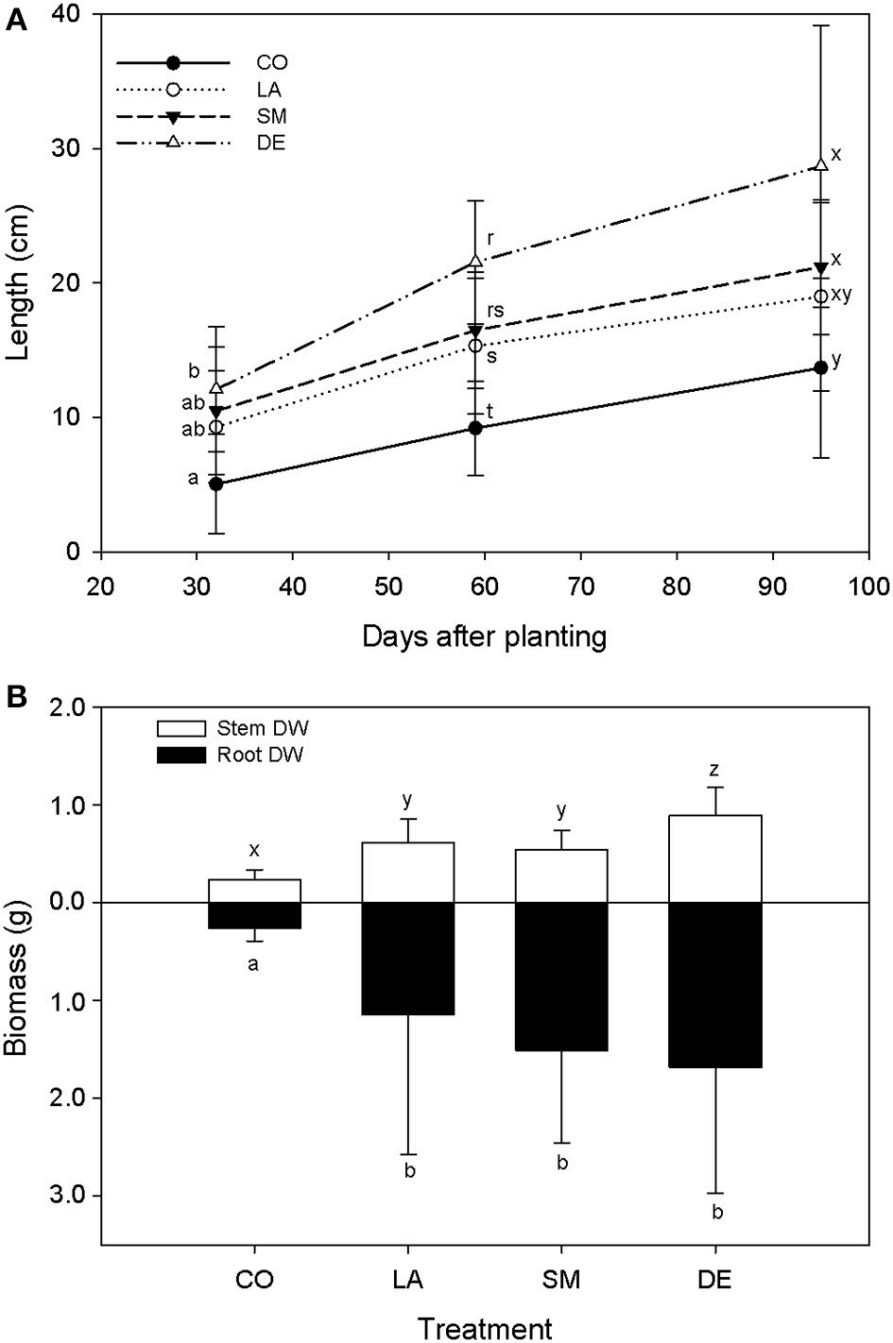
**Willow growth throughout the experiment (A) and willow biomass at TF (B) for CO, DE, SM, and LA treatments**. Error bars represent standard deviation.

All 40 willows planted in the contaminated soil survived over the 100 days of the experiment. However, CO willows showed delayed growth, and were smaller throughout the experiment than those that had been inoculated (Figure 2A). DE willows had significantly longer stems (*P* < 0.05) than the CO willows throughout the experiment, while LA willows were only significantly taller (*P* < 0.05) at days 59 and 95, and SM willows treatment were only significantly taller (*P* < 0.05) at day 59 (Figure 2A). Furthermore, at day 59, the stems of DE willows were significantly longer (*P* < 0.05) than those from SM willows (Figure 2A). At the end of the experiment (TF), there was a significant effect (*P* < 0.05) of inoculation on shoot and root biomass, with significant differences (*P* < 0.05) between the non-inoculated controls (CO) and the inoculated treatments (DE, SM, and LA; Figure 2B). Within the inoculated treatments, the DE willows produced significantly more shoot biomass (*P* < 0.05), while root biomass production was comparable across the three inoculation treatments (Figure 2B). Willows planted in parallel in non-contaminated potting soil were on average 96.6 cm high, with an average root biomass of 2.81 g and an average shoot biomass of 14.92 g at TF. DNA yields from soil were on average 1.80 μg per g soil for T0 soils, 4.30 μg per g soil for the three inocula, and 5.14 μg per g soil for TF soils (Figure 1). There was a significant difference in DNA yields between T0 and TF samples (repeated-measure ANOVA: *F* = 89.84, *P* < 0.001), but no significant differences were observed between treatments or for the interaction term (*P* > 0.05).

### Archaeal community

**Figure 3:**
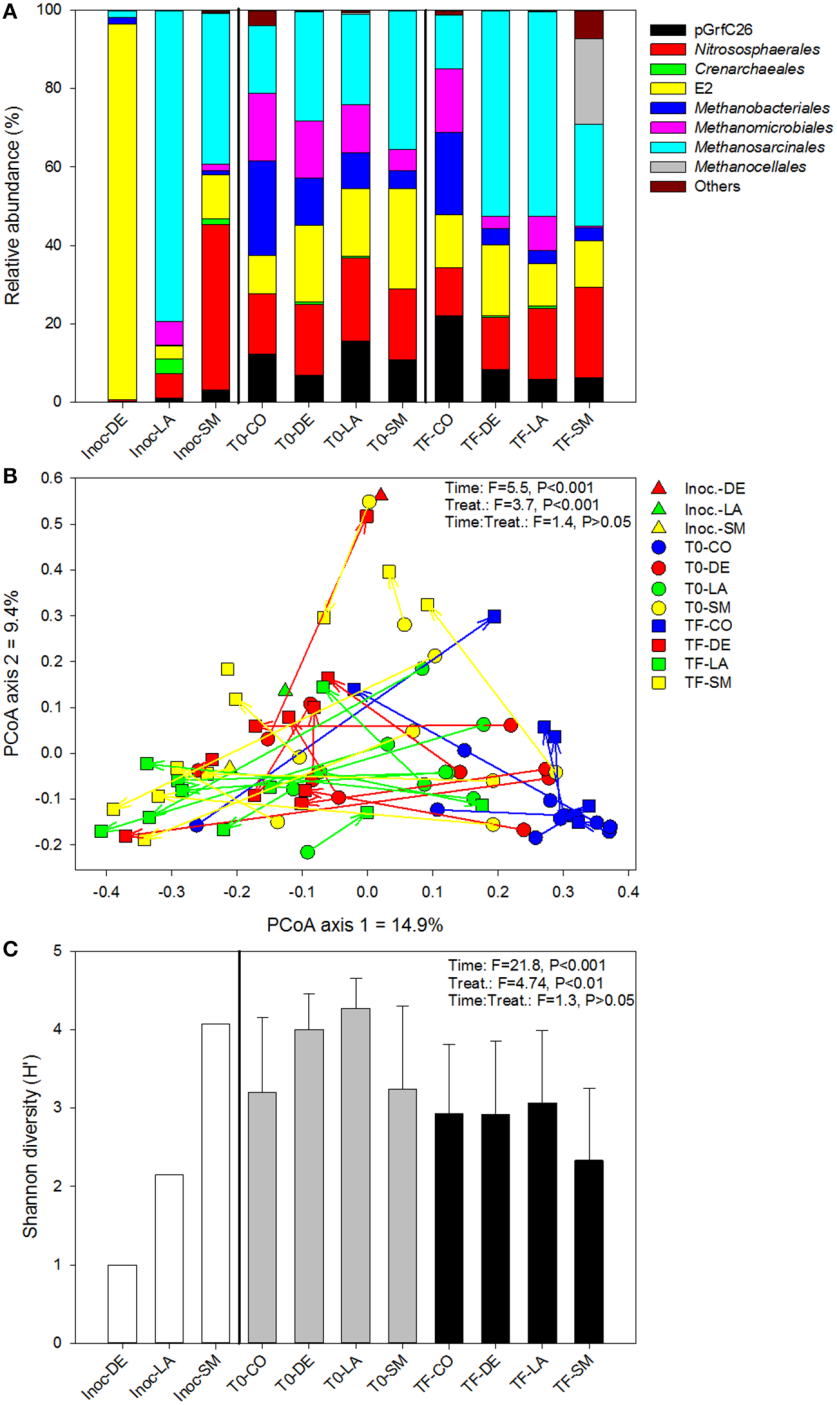
**Archaeal community composition (A), structure (B), and diversity (C) for bulk and willow rhizosphere soil samples taken at T0 and TF (after 100 days) for CO, DE, SM, and LA treatments and the original inocula**. Vectors are linking samples taken from the same pot at different time points. Error bars represent standard deviation.

At the order level, the archaeal community was very different across inoculum types, with a dominance of the E2 group in the DE inoculum, the *Methanosarcinales* in the LA inoculum, and the *Nitrososphaerales* in the SM inoculum (Figure 3A). At T0 (after mixing the inocula with the irradiated soil), the three inoculated treatments were more similar, being co-dominated by *Nitrososphaerales, Methanobacteriales*, E2 group, *Methanosarcinales*, and *Methanomicrobiales* (Figure 3A). At TF, the CO soils did not differ markedly from their T0 counterparts, while the DE and LA soils showed an increased dominance of the *Methanosarcinales* and the SM soils showed increased dominance by the *Methanocellales* (Figure 3A). The ordination resulting from principal coordinate analysis of Bray–Curtis distances based on OTU relative abundance showed high variability within each of the treatments, especially within the LA and SM rhizospheric soils, while communities from the CO treatment generally clustered together (Figure 3B). However, inoculation appeared to have some influence, as the TF inoculated samples generally clustered on the left side of the ordination, and for the SM and LA treatments, the TF samples mostly clustered toward their initial inoculum (Figure 3B). Time and treatment had similar effects (similar *F*-ratios) in permanova tests (Figure 3B), and when separating T0 and TF samples, the effect of treatment was stronger at TF (Table 1). Permanova tests also revealed significant relationships between shoot biomass and archaeal community structure. A slightly stronger link was observed between shoot biomass and the TF community (higher *F*-ratio) (Table 1). Diversity was lower in the DE and LA inocula when compared to the SM inoculum (Figure 3C). Repeated-measure ANOVA tests demonstrated that archaeal diversity was significantly influenced by treatment type, an effect that was mainly driven by significant differences between the CO, SM, and LA treatments (Figure 3C). There was also a significant effect of time on archaeal diversity, with lower diversity in TF samples for all treatments (Figure 3C). The initial archaeal diversity (at T0) was significantly correlated with shoot biomass (*r*_*s*_ = 0.321, *P* = 0.049), but not root biomass, and no correlations were significant for diversity at TF. Some of the archaeal genera identified at T0 or TF had significant positive or negative correlations with willow biomass (Table 2). The relative abundance of *Methanosarcina* at T0 was significantly and positively correlated to root and shoot biomass, while its relative abundance at TF was significantly and positively correlated to root biomass (Table 2). Other genera also showed significant correlations with shoot and root biomass and are listed in Table 2.

**Table 1:** **Permanova analysis**.

**Time**	**Factor**	***F*-ratio**	***P*-values**
**ARCHAEA**			
T0	Treatment	2.18	0.001
T0	Root biomass	1.73	0.065
T0	Shoot biomass	2.49	0.009
TF	Treatment	3.62	0.001
TF	Root biomass	1.45	0.185
TF	Shoot biomass	3.05	0.017
**BACTERIA**			
T0	Treatment	17.61	0.001
T0	Root biomass	4.71	0.006
T0	Shoot biomass	8.50	0.002
TF	Treatment	4.35	0.001
TF	Root biomass	3.03	0.004
TF	Shoot biomass	4.07	0.001
**FUNGI**			
T0	Treatment	7.40	0.001
T0	Root biomass	2.33	0.011
T0	Shoot biomass	4.45	0.001
TF	Treatment	2.87	0.001
TF	Root biomass	1.73	0.012
TF	Shoot biomass	2.72	0.001

**Table 2:** **Significant Spearman correlations between the relative abundance of archaeal genera and root or shoot biomass**.

**Genus**	**Time**	***r_*s*_***	***P*-values**
**ROOT BIOMASS—POSITIVE**			
*Methanosarcina*	T0	0.400	0.0127
SAGMA group	TF	0.369	0.0228
*Methanosarcina*	TF	0.359	0.0268
**ROOT BIOMASS—NEGATIVE**			
Unid. *Methanomicrobiales*	TF	−0.519	0.0008
*Methanobacterium*	TF	−0.399	0.0132
pGrfC26	T0	−0.323	0.0476
**SHOOT BIOMASS**—**POSITIVE**			
SAGMA group	TF	0.406	0.0114
*Methanosarcina*	T0	0.381	0.0183
MCGCL group	T0	0.375	0.0204
*Methanomassiliicoccus*	TF	0.350	0.0312
**SHOOT BIOMASS—NEGATIVE**			
Unid. *Methanomicrobiales*	TF	−0.515	0.0009
*Methanobacterium*	T0	−0.390	0.0154
Unid. *Methanomicrobiales*	T0	−0.342	0.0354

*Unid., unidentified; OTU in the Greengenes database that was not identified at the genus level. The next lowest taxonomical level for which the OTU was identified is given*.

### Bacterial community

**Figure 4:**
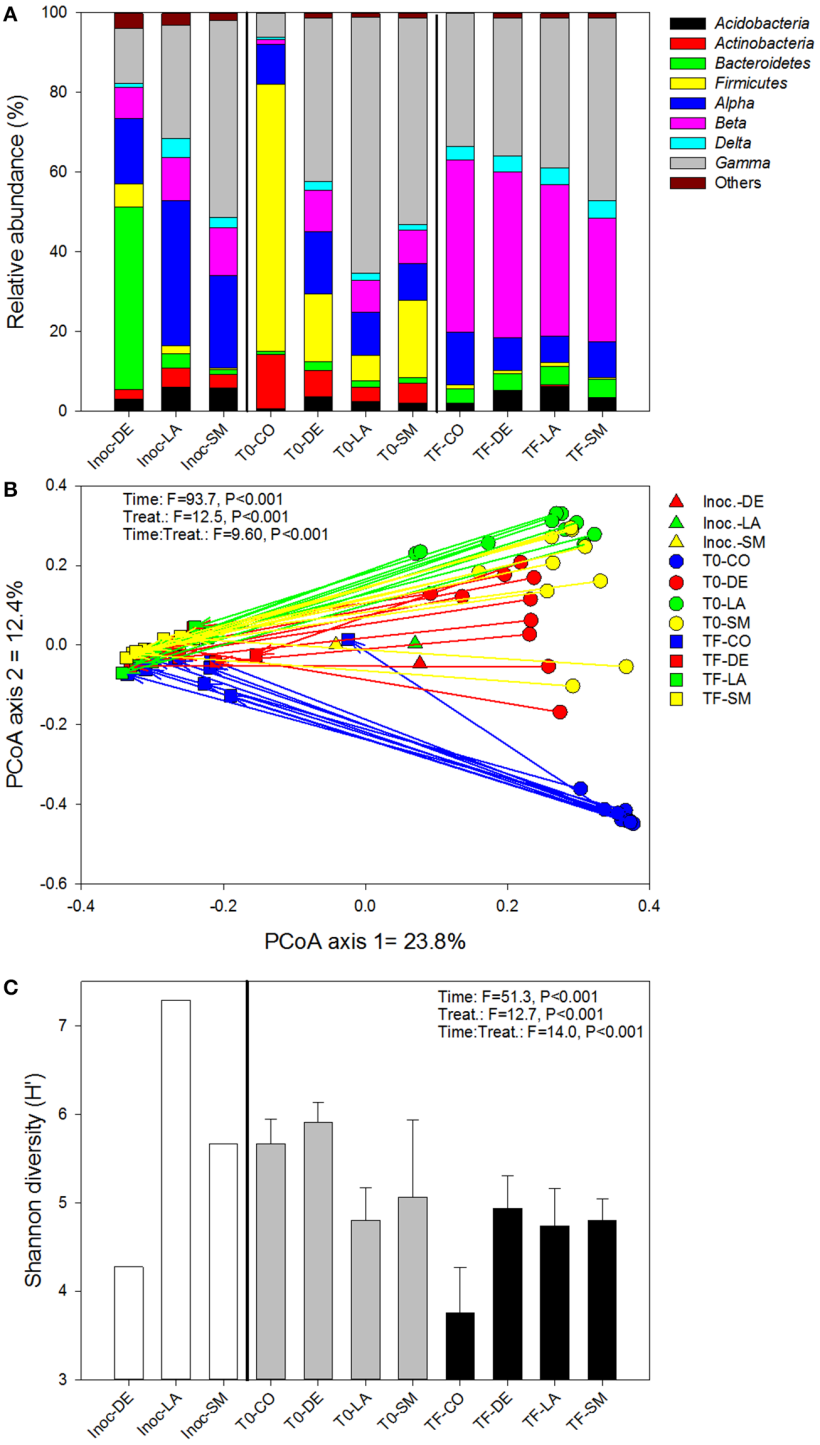
**Bacterial community composition (A), structure (B), and diversity (C) for bulk and willow rhizosphere soil samples taken at T0 and TF (after 100 days) for CO, DE, SM, and LA treatments and the original inocula**. Vectors are linking samples taken from the same pot at different time points. Error bars represent standard deviation.

The bacterial inocula showed marked differences, with the inocula originating from rhizospheric soil (LA and SM treatments) dominated by *Alpha*-, *Beta*-, and *Gammaproteobacteria*, while the inoculum originating from bulk soil (DE treatment) was dominated by *Bacteroidetes*, with the *Firmicutes, Alpha*-, *Beta*-, and *Gammaproteobacteria* present at moderate abundance (Figure 4A). The bacterial communities remained variable at T0, with a large dominance of *Firmicutes* in the CO treatment and a dominance of *Proteobacteria* (mainly *Gammaproteobacteria*) in the inoculated treatments (DE, SM, and LA; Figure 4A). After 100 days of growth (TF), the bacterial community composition of the willow rhizosphere was remarkably similar between all treatments, with a co-dominance of *Beta*- and *Gammaproteobacteria* (Figure 4A). This convergence of the bacterial communities at TF was also visible in the PCoA ordination of Bray–Curtis distances, in which the communities are dispersed at T0 and much more similar at TF (Figure 4B). Bacterial communities in the willow rhizosphere at TF were not especially similar to the bacterial communities of the initial inocula (Figure 4B). Permanova showed that time was the major factor leading to differences in bacterial composition (highest *F*-ratio), but there were also highly significant effects of the treatments and of the interaction term. When separating the T0 and TF samples, the effect of treatment was significant for both datasets, although the *F*-ratio was much larger for the T0 dataset (Table 1). This was also visible in the ordinations. There was also a significant relationship between the bacterial community structure at T0 and TF and root and shoot biomass in permanova tests, with a stronger effect for T0 (higher *F*-ratios; Table 1). Bacterial diversity was significantly affected by time, treatment, and the interaction term (Figure 4C). Diversity was largest in the CO and DE treatments at T0 and was at its lowest in the CO rhizosphere at TF (Figure 4C). Bacterial diversity at T0 was not significantly correlated to root and shoot biomass, but significant correlations were observed at TF between bacterial diversity, shoot biomass (*r*_*s*_ = 0.650, *P* < 0.001), and root biomass (*r*_*s*_ = 0.669, *P* < 0.001). A variety of bacterial genera showed significant correlations with root and shoot biomass, and the top 10 strongest positive and negative correlations are presented in Table 3. Some of the correlations were very strong, with *P*-values well below 1 × 10^−5^. Among the most significant positive correlations, many of the identified taxa have previously been reported to be associated with plants.

**Table 3:** **Top 10 most significant Spearman correlations between bacterial genera relative abundance and root or shoot biomass**.

**Genus**	**Time**	***r_*s*_***	***P*-values**
**ROOT BIOMASS—POSITIVE**			
Unid. *Alphaproteobacteria*	T0	0.738	5.44 × 10^−8^
Unid. *Cytophagaceae*	TF	0.693	7.20 × 10^−7^
Unid. *Oxalobacteraceae*	T0	0.658	4.03 × 10^−6^
Unid. *Alcaligenaceae*	T0	0.634	1.14 × 10^−5^
*Aquicella*	T0	0.593	5.49 × 10^−5^
Unid. *Sinobacteraceae*	TF	0.580	8.83 × 10^−5^
Unid. *Ellin329*	TF	0.572	0.000114
Unid. *Betaproteobacteria*	TF	0.568	0.000131
Unid. *DS.18*	T0	0.554	0.000208
Unid. *Alphaproteobacteria*	TF	0.553	0.000217
**ROOT BIOMASS—NEGATIVE**			
Unid. *OPB41*	T0	−0.585	7.45 × 10^−5^
Unid. *Bacillales*	T0	−0.578	9.23 × 10^−5^
Unid. *Solirubrobacterales*	T0	−0.568	0.000130
*Cohnella*	T0	−0.549	0.000243
*Pseudoxanthomonas*	TF	−0.548	0.000252
*Brevibacillus*	T0	−0.540	0.000326
*Pedobacter*	TF	−0.507	0.000835
*Ammoniphilus*	T0	−0.502	0.000966
*Paenibacillus*	T0	−0.502	0.000976
*Sporosarcina*	T0	−0.500	0.00102
**SHOOT BIOMASS—POSITIVE**			
*HB2.32.21*	TF	0.683	1.20 × 10^−6^
Unid. *Xanthomonadaceae*	T0	0.678	1.56 × 10^−6^
Unid. *Betaproteobacteria*	TF	0.667	2.57 × 10^−6^
Unid. *Coxiellaceae*	T0	0.635	1.06 × 10^−5^
Unid. *Alphaproteobacteria*	T0	0.621	1.94 × 10^−5^
Unid. *Ellin6067*	TF	0.605	3.59 × 10^−5^
Unid. *Comamonadaceae*	T0	0.590	6.17 × 10^−5^
*Aquicella*	T0	0.589	6.48 × 10^−5^
Unid. *Solibacteraceae*	T0	0.585	7.47 × 10^−5^
*Leptothrix*	TF	0.584	7.68 × 10^−5^
**SHOOT BIOMASS—NEGATIVE**			
Unid. *Bacillales*	T0	−0.627	1.51 × 10^−5^
Unid. *Peptostreptococcaceae*	T0	−0.597	4.75 × 10^−5^
Unid. *Planococcaceae*	T0	−0.567	0.000137
Unid. *Gracilibacteraceae*	T0	−0.561	0.000166
*Pilimelia*	T0	−0.525	0.000512
*Solibacillus*	T0	−0.524	0.000523
Unid. *Acidimicrobiales*	T0	−0.523	0.000542
*Turicibacter*	T0	−0.522	0.000545
Unid. *Thermoactinomycetaceae*	T0	−0.519	0.000596
Unid. *Ruminococcaceae*	T0	−0.516	0.000649

*Unid., unidentified; OTU in the Greengenes database that was not identified at the genus level. The next lowest taxonomical level for which the OTU was identified is given*.

### Fungal community

**Figure 5:**
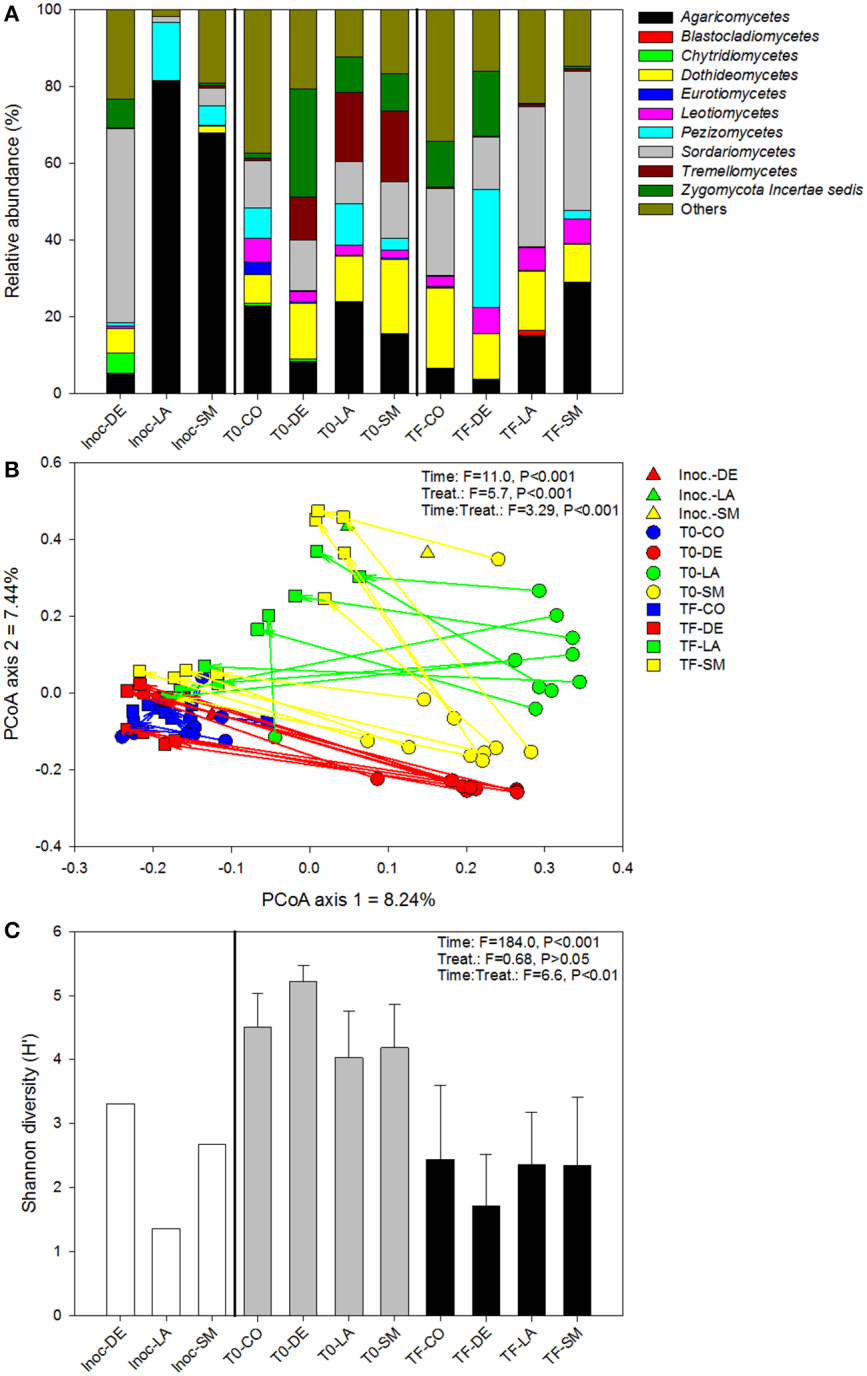
**Fungal community composition (A), structure (B), and diversity (C) for bulk and willow rhizosphere soil samples taken at T0 and TF (after 100 days) for CO, DE, SM, and LA treatments and the original inocula**. Vectors are linking samples taken from the same pot at different time points. Error bars represent standard deviation.

The DE inoculum differed markedly from the rhizospheric inocula (LA and SM), harboring relatively more *Sordariomycetes, Dothideomycetes, Chytridiomycetes*, and *Zygomycota*, and relatively less *Agaricomycetes* and *Pezizomycetes* (Figure 5A). Differences between treatments were also visible at T0 and TF, with the CO and DE treatments differing substantially from the LA and SM treatments (Figure 5A). Large differences in the dominant class were visible between sampling points and treatments, with the *Agaricomycetes, Dothideomycetes, Pezizomycetes, Sordariomycetes, Tremellomycetes*, and *Zygomycota* dominating or co-dominating the various treatments (Figure 5A). In the ordination based on principal coordinates analysis of Bray–Curtis distances of OTU tables, a similar story emerged (Figure 5B). At T0, the four treatments were clearly distinct in the ordination space, with a few outliers (Figure 5B). The rhizospheric (SM and LA) inocula and the DE inoculum were also clearly separated in the ordination space (Figure 5B). Some of the fungal communities at TF converged toward their respective inocula, with 5/10 samples for the SM treatment, 5/10 samples for the LA treatment, and 10/10 samples for the DE treatment (Figure 5B). The LA and SM samples that did not converge toward their initial inoculum and all the DE samples grouped together with the CO samples at TF (Figure 5B). The CO treatment did not change much through the course of the experiment and samples from T0 and TF were located together in the ordination space (Figure 5B). The large effect of the inoculation treatments resulted in a smaller difference between the *F*-ratio for the effect of time and treatment in permanova tests as compared to bacteria and archaea. Separate permanova tests for the effect of treatment on T0 and TF communities revealed highly significant effects, with stronger effects (higher *F*-ratio) for the T0 communities (Table 1). Similarly, the relationship between root and shoot biomass and fungal community structure was stronger (higher *F*-ratio) for T0 communities than TF communities (Table 1). In terms of diversity, there was a significant effect of time, with significantly higher diversity in T0 samples than TF samples for all treatments (Figure 5C). The interaction term was also significant in ANOVA tests, which was due to the fact that the differences in diversity between treatments observed at T0 were no longer visible at TF (Figure 5C). Fungal diversity at T0 was not significantly correlated with root or shoot biomass (*P* > 0.05), but there was a significant negative correlation between fungal diversity at TF and shoot biomass (*r*_*s*_ = −0.322, *P* = 0.045). The relative abundances of individual genera were also tested for correlation with willow biomass, and the 10 strongest positive and negative correlations are reported in Table 4. Most of the strongest positive correlations with willow biomass were fungal genera at T0, while the strongest negative correlations were with fungal genera at TF or T0 (Table 4). *Sphaerosporella* showed a particular pattern at TF; it was nearly absent in most samples (0–2.7%), but extremely abundant (58.2–93.7%) in the rhizosphere of the four willows that showed the highest shoot biomass (all from the DE treatment). This resulted in a significant positive Spearman correlation with shoot biomass (Table 4).

**Table 4:** **Top 10 most significant Spearman correlations between fungal genera relative abundance and root or shoot biomass**.

**Genus**	**Time**	***r_*s*_***	***P*-values**
**ROOT BIOMASS—POSITIVE**			
*Mortierella*	T0	0.646	6.75 × 10^−6^
*Epicoccum*	T0	0.628	1.44 × 10^−5^
*Pseudogymnoascus*	T0	0.601	4.15 × 10^−5^
*Valsa*	T0	0.579	9.20 × 10^−5^
*Sporidiobolus*	T0	0.481	0.0017
*Rosellinia*	T0	0.475	0.0019
*Cosmospora*	TF	0.465	0.0029
*Cystofilobasidium*	T0	0.462	0.0027
*Cosmospora*	T0	0.441	0.0044
*Cryptococcus*	T0	0.418	0.0073
**ROOT BIOMASS—NEGATIVE**			
*Pseudaegerita*	T0	−0.613	2.60 × 10^−5^
*Cladosporium*	TF	−0.529	0.0005
*Byssochlamys*	T0	−0.483	0.0016
*Ganoderma*	T0	−0.456	0.0031
*Talaromyces*	T0	−0.414	0.0079
*Rhizophagus*	T0	−0.364	0.0209
*Eupenicillium*	TF	−0.351	0.0284
*Blastocladiella*	TF	−0.349	0.0293
*Lignincola*	T0	−0.338	0.0330
*Pseudogymnoascus*	TF	−0.327	0.0419
**SHOOT BIOMASS—POSITIVE**			
*Pseudogymnoascus*	T0	0.636	1.04 × 10^−5^
*Curvularia*	T0	0.529	0.0004
*Udeniomyces*	T0	0.505	0.0009
*Mortierella*	T0	0.499	0.0010
*Trichocladium*	T0	0.484	0.0015
*Sphaerosporella*	TF	0.476	0.0022
*Clonostachys*	T0	0.472	0.0021
*Epicoccum*	T0	0.470	0.0022
*Mrakia*	T0	0.438	0.0047
*Leucosporidium*	T0	0.431	0.0055
**SHOOT BIOMASS—NEGATIVE**			
*Cladosporium*	TF	−0.403	0.011
*Geotrichum*	T0	−0.382	0.015
*Hebeloma*	T0	−0.378	0.016
*Talaromyces*	TF	−0.351	0.028
*Rhizophagus*	T0	−0.348	0.028
*Pseudogymnoascus*	TF	−0.346	0.031
*Ganoderma*	T0	−0.346	0.029
*Occultifur*	T0	−0.346	0.029
*Eupenicillium*	TF	−0.335	0.037
*Setoseptoria*	T0	−0.317	0.046

## Discussion

The microbiome of contaminated soils was successfully modified by gamma-irradiation followed by the introduction of various soil inocula. Bacterial and fungal communities from the four treatments were clearly distinct at the beginning of the experiment (T0), with respect to both microbial community composition and diversity. However, after 100 days of willow growth (TF), the original differences were not visible for most treatments, with the exception of the fungal communities for some samples. This convergence of the willow rhizosphere microbiome at TF suggests that the willow rapidly exerts strong selective pressures in the rhizosphere, selecting for a similar microbiome from variable starting microbiomes. This strong selective environment has been reported for other plant species, and resulted in sharp contrasts between the microbial community composition of the rhizosphere and adjacent bulk soil (Smalla et al., 2001; Kowalchuk et al., 2002; Griffiths et al., 2006; Kielak et al., 2008; Bulgarelli et al., 2012; Peiffer et al., 2013). This selective pressure often varies between plant species (Haichar et al., 2008; Berg and Smalla, 2009) and even genotypes (Lundberg et al., 2012; Sugiyama et al., 2012). Here, we observed a relatively low variability in microbiome composition between individual willows possibly because we used a clonal population of willows. This could partly explain the striking convergence in willow rhizosphere communities at TF. For willows planted in contaminated soils, this selection pressure was previously shown to result in an increased expression of microbial genes related to the degradation of hydrocarbons, as well as large shifts in the active microbial community relative to willows planted in non-contaminated soil or contaminated bulk soil (Yergeau et al., 2014; Pagé et al., 2005). Because of this overwhelming rhizosphere effect, the inoculation of a pre-selected microbiome was only effective in the short term, even though we had disrupted the indigenous soil's microbiome using irradiation. Although, the experimental treatments did not produce lasting microbiome modifications, significant changes were observed in willow biomass production at TF. Many of our results suggest that microbial community composition at TF was a poor indicator of willow growth and biomass production compared with community composition at T0. Thus, the strategy of using irradiation to reduce the microbial load and open niches for microbial colonization successfully modified the starting microbiome of contaminated soil, which led to lasting differences in willow growth.

Our hypothesis was that willows growing in pots inoculated with rhizospheric soil harvested from willows that had grown successfully in contaminated soils would grow more successfully than willows receiving other inoculants. In contrast to our hypothesis, willows growing in soil inoculated with bulk soil (DE treatment) performed better than those growing in pots inoculated with rhizospheric soil (LA and SM). There were no apparent differences in survival rates, as all willows survived throughout the length of the experiment. One possible explanation for the better performance of the DE treatment is that the DE inoculum was in fact bulk soil (since the willow had died) as compared to rhizospheric soil for the LA and SM inocula. As mentioned above, the rhizosphere is a strongly selective environment, which promotes a lower diversity of specialized microorganisms (Marilley and Aragno, 1999), and in fact at T0, the DE pots were more diverse in terms of the bacteria and fungi present. The willows growing in these pots were exposed to a wider diversity of organisms, which may have helped them to initially adapt to the stressful conditions created by the contaminants.

Although, the soil used to pot the second-generation willows was harvested at the exact same location as the soil used for the study that produced the first-generation willows, it should be stressed that the experimental conditions in this study were different: smaller pots, greenhouse vs. outdoor incubation, willows directly planted in soil vs. pre-growth followed by transplantation of clippings, winter vs. spring, irradiated soil vs. fresh soil. This probably explains the observed differences in survival rates, with all the second-generation willows surviving compared to an 89% mortality rate for the first-generation willows. Willows survival in contaminated soil was previously shown to differ markedly depending on field environmental conditions (Guidi et al., 2011). The different experimental conditions also likely modified the rhizosphere–willow association, as well as the composition of the ideal microbiome that would allow optimal growth. Alternatively, rhizosphere communities are known to change over time (Chaparro et al., 2013b) because of shifts in plant exudates (Chaparro et al., 2013a), and the rhizosphere communities that were harvested and used as inocula (6 month old plants) were probably not optimal for willow clipping establishment in soil. Access to a more diverse microbiome might have given an advantage to the DE willows by allowing them to select the best microbiome for the growth conditions and their developmental stage. This suggests a very high specificity of rhizosphere–willow associations under stressful conditions, but a high variability in the composition of the optimal microbiome, depending on growth conditions and plant developmental stage. This further complicates efficient engineering of a beneficial microbiome.

The willows from the CO treatment showed reduced growth and distinct starting microbial communities compared to the willows from other treatments. One possible explanation could be that certain key microbes required for efficient willow establishment and growth in highly contaminated environments were killed by the irradiation treatment, and could not be recruited in the CO treatment because of the lack of inoculation. Alternatively, some deleterious organisms may have survived irradiation, and rapidly colonized newly available niches. Correlation analyses highlighted some of the potentially beneficial and deleterious organisms that were highly correlated to willow biomass. Consequently, instead of trying to modify whole microbial communities, an alternative approach would be to ensure that beneficial species are present in high abundance, while restricting the abundance of deleterious taxa. Soil microorganisms can have large effects on plant growth and function (Hoeksema et al., 2010; Glassman and Casper, 2012; Lau and Lennon, 2012), although the relative impact of beneficial and deleterious microorganisms will differ depending on soil type, environmental conditions, and plant species. For instance, mycorrhizal fungi are generally beneficial, but can become parasitic under certain environmental conditions, especially in human-managed ecosystems (Johnson et al., 1997). Accordingly, we found a negative correlation between the mycorrhizal fungi genus *Rhizophagus* and shoot biomass. Alternatively, the correlations between willow biomass and microbial relative abundance could be indirect, through the effect of the microorganisms on other soil organisms or soil physico-chemical characteristics.

The lack of inoculation in the CO treatment also resulted in significantly lower bacterial diversity in the willow rhizosphere at TF. In fact, bacterial diversity at TF was strongly and positively correlated to willow biomass. High community evenness and diversity have been shown to result in healthy soils, high levels of nutrient cycling, increased plant productivity, and reduced stress and disease incidence (Elliot and Lynch, 1994; van Bruggen and Semenov, 2000; Wittebolle et al., 2009; Crowder et al., 2010). A lack of plant community evenness has been associated with reduced plant productivity, possibly due to niches being left vacant and the loss of certain ecosystem services (Wilsey and Potvin, 2000). One way to optimize the willow microbiome might be to provide a soil bacterial community with high diversity and evenness, to allow the willow to select its preferred rhizosphere organisms for optimal growth. This may help to avoid pressures that could lead to selection of suboptimal communities, such as microbial priority effects. However, polluted environments rarely contain diverse or even microbiomes, and one key to effective phytoremediation may be to restore soil bacterial evenness by, for example, soil fertilization, mixing, or aeration before the introduction of plants. Indicative of the importance of restoring soil quality, the willows planted in the contaminated soils only grew to a fraction of the size of those that were grown in parallel in nutrient-rich, well-aerated potting media.

In contrast to bacteria and archaea, the diversity of fungi showed a weak but significant negative correlation with willow shoot biomass and some fungal communities had converged toward the composition of their respective inocula by the end of the experiment. The difference between fungal and bacterial and archaeal communities could be due to the more intimate nature of the relationship between fungi and plants, as many obligate symbionts and pathogens of plants are found in the fungal domain. Previous studies of willows growing in contaminated soil highlighted the stronger link between fungal communities and willow cultivar identity (Bell et al., 2014a) and between fungi and willow growth and zinc uptake (Bell et al., 2015) as compared to bacterial communities. Fungal diversity was also enhanced significantly more by willow introduction than was bacterial diversity, suggesting that phytoremediation may have a disproportionate direct effect on fungi (Bell et al., 2014a). Fungi and bacteria can also be antagonists in the soil environment (De Boer et al., 2005; Rousk et al., 2008; Bonfante and Anca, 2009; Schrey et al., 2012), and competition between these groups has been shown to reduce key soil functions (Siciliano et al., 2009) and microbial growth (Mille-Lindblom et al., 2006; Meidute et al., 2008). Taken together, these results indicate that the physiological and ecological differences between fungi and bacteria may require domain-specific microbiome engineering strategies.

The relative abundance of certain genera at T0 appeared to play a key role in willow growth. For many of the genera (especially fungi) with the strongest positive correlations with willow growth, it was often their relative abundance at T0 that was most strongly correlated to final willow characteristics. Furthermore, the microbial communities of the different treatments were often dissimilar at T0, closely mirroring eventual differences in willow growth, while the TF communities were more similar to each other, and less strongly related to differences in willow growth. This data strongly implies that the microbiome composition at T0 plays a role in determining eventual willow growth in stressful environments. This is in line with our recent results that show that willow growth and Zn accumulation after 16 months of growth in the field were more strongly related to the abundance of the ectomycorrhizal fungus *Sphaerosporella brunnea* at 4 months than to its abundance at 16 months (Bell et al., 2015).

## Conclusions

Modifying the soil microbiome through gamma-irradiation followed by soil inoculation resulted in short-term shifts in microbial communities, but lasting effects on plant growth characteristics. Our study demonstrated the potential for modifying target plant characteristics through manipulation of the plant-associated microbiome, even though this did not occur as we had hypothesized. This study also highlights several key factors that should be considered when engineering the plant rhizosphere microbiome, including the presence and abundance of keystone species, diversity and evenness of the initial inoculum, ecological differences between fungi and bacteria, environmental conditions, and the plant growth stage that the inoculum originates from.

### Conflict of interest statement

The authors declare that the research was conducted in the absence of any commercial or financial relationships that could be construed as a potential conflict of interest.
